# Extracorporeal Shock Wave Stimulation as Alternative Treatment Modality for Wrist and Fingers Spasticity in Poststroke Patients: A Prospective, Open-Label, Preliminary Clinical Trial

**DOI:** 10.1155/2016/4648101

**Published:** 2016-07-18

**Authors:** Robert Dymarek, Jakub Taradaj, Joanna Rosińczuk

**Affiliations:** ^1^Department of Nervous System Diseases, Faculty of Health Science, Wrocław Medical University, Bartla 5 Street, 51-618 Wrocław, Poland; ^2^Department of Physiotherapy Basics, Academy School of Physical Education in Katowice, Mikolowska 65 Street, 40-065 Katowice, Poland

## Abstract

*Objective.* To evaluate the effectiveness of radial shock waves (rESW) for wrist and fingers flexors spasticity in stroke patients.* Methods.* Twenty patients with upper limb muscle spasticity were enrolled in the study and treated with a single session of rESW. The spasticity level of the radio carpal (RC) and finger (FF) joints was assessed using Modified Ashworth Scale (MAS). The resting bioelectrical activity of the flexor carpi radialis (FCR) and flexor carpi ulnaris (FCU) was examined using surface electromyography (sEMG). Trophic conditions were measured using infrared thermal (IRT) imaging. All measurements were conducted at baseline (*t*_0_), immediately after rESW (*t*_1_), and 1 (*t*_2_) and 24 (*t*_3_) hours following rESW.* Results.* Significant reduction in MAS was observed for the RC joint in *t*_1_, as well as for the FF joints in *t*_1_, *t*_2_, and *t*_3_. A significant decrease in sEMG was shown for the FCR muscle in *t*_1_ and *t*_2_, as well as for the FCU muscle in *t*_1_ and *t*_3_. Also, a significant increase in IRT value was observed in *t*_3_ only.* Conclusions.* A single session of rESW could be an effective alternative treatment for reduction of limb spasticity and could lead to improvement of trophic conditions of the spastic muscles.

## 1. Introduction

Stroke is a sudden episode defined as a neurological deficit of vascular aetiology attributed to an acute focal injury of the central nervous system (CNS). This can be due to cerebral infarction, well known as ischaemic stroke, or haemorrhagic stroke in the form of intracerebral and subarachnoid haemorrhage [1, 2].

CNS infarction is characterized as brain (or spinal cord) cell death attributable to ischaemia, based on neuropathological, neuroimaging, and/or clinical evidence of permanent injury [3]. It was determined that higher rates of stroke were observed in eastern European countries and lower rates were observed in southern European countries. Differences in total stroke incidence among the participating European centres are mainly attributed to variations in the risk of ischaemic stroke (less variation was observed for haemorrhagic stroke) [4, 5].

One of the signs of CNS damage after stroke is muscle spasticity, which is caused by an upper motor neuron (UMN) lesion. Spasticity, according to Lance's definition, “is a motor disorder characterized by a velocity dependent increase in tonic stretch reflexes (muscle tone), with exaggerated tendon jerks, resulting from hyperexcitability of the stretch reflexes, as one component of the UMN syndrome” [6].

It has been demonstrated that 12 million people worldwide suffer from spastic paresis of the upper or lower limb [7, 8]. It is proved that, 1 year after stroke, the number of patients demonstrating spasticity increases rapidly to almost 40% or even 70% [9]. Spasticity further interferes with important functions of daily living related to long-term complications, such as chronic pain, deformities of joints, heterotrophic ossification of soft tissues, demineralization of bones, contractures of muscles and their subsequent atrophy, and blood circulation disturbances [10, 11].

There is a wide range of procedures for the management of spasticity including antispasticity medication, intrathecal baclofen, phenol and ethanol injections, administration of botulinum toxin, and surgical interventions [12, 13]. In addition, physiotherapy modalities, including physical therapy methods, such as neuromuscular stimulation (NMS), electromyography-triggered neuromuscular stimulation (EMG-NMS), transcutaneous electrical nerve stimulation (TENS), functional electrical stimulation (FES), or ultrasound therapy (UT), are commonly used [14, 15]. One of the promising methods nowadays, but still not widely used in everyday practice, for spasticity reduction is extracorporeal shock wave (ESW).

Recent studies have indicated that ESW treatment is a quite novel physical modality, which shows a valuable potential, promising effect, and suitable safety in the treatment of spasticity caused by UMN lesion [16, 17]. Significant effectiveness was observed in patients suffering from different neurological pathologies, such as cerebral palsy [18–20], multiple sclerosis [21], and, in particular, stroke [22–29].

In spite of these important preliminary findings, the mechanism of ESW action, especially radial ESW (rESW), is still under investigation and further well-designed studies are still in demand. It should be pointed out that the rESW pneumatic generator was implemented in upper limb spasticity in only one study conducted by Daliri et al. in 2015 [22], who showed beneficial results with this type of ESW in the treatment of upper limb spasticity after stroke. However, to the best of our knowledge, at present, no papers have observed the resting bioelectrical activity or the temperature distribution of the treated spastic muscles.

The main purpose of the present study was to evaluate the short-term effectiveness of a single session of rESW applied to the spastic wrist and fingers flexor muscles in poststroke patients. The clinical evaluation was conducted with the objective observation of the chosen electrophysiological and thermographic parameters.

## 2. Methods

### 2.1. Ethical Approval and Design

This study is a preliminary, prospective, open-label, clinical trial to assess the effect of rESW in poststroke spasticity of the upper limb. The study was conducted according to the Declarations of Helsinki and the guidelines for Good Clinical Practice. The study protocol was approved by the Bioethics Committee of the Wrocław Medical University (number KB–610/2012). The project was funded by the Polish National Science Centre (number DEC–2011/03/N/NZ7/00327).

### 2.2. Participants

#### 2.2.1. Inclusion Criteria

**Figure 1:**
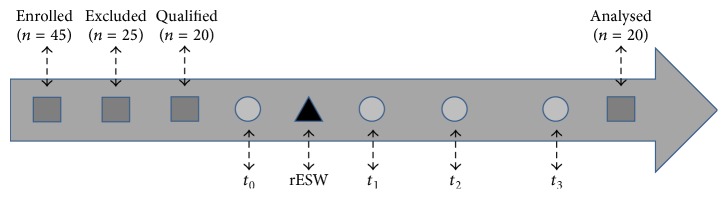
Timeframe of patients' qualification procedure with outlined rESW intervention and measurement time points (*t*_0_, *t*_1_, *t*_2_, and *t*_3_).

Patients were enrolled at the Department of Neurological Rehabilitation of the Provincial Specialist Hospital in Wrocław, Poland. The inclusion criteria at screening and at the baseline visit were as follows: (1) patients must be aged at least 18 years, but not older than 80 years; (2) patients must have had a first-ever episode of ischaemic stroke in their medical history; (3) the onset of ischaemic stroke must be at least 9 months previously (4) but not longer than 10 years ago (chronic phase); (5) spasticity of the wrist and fingers' flexor muscles must be assessed by 1+ score using the Modified Ashworth Scale (MAS); (6) there must be a lack of adjunctive interventions reducing spasticity during this study (botulinum toxin, phenol, or alcohol injections); (7) there must be a lack of contraindications to undergoing ESW stimulation (coagulation disorders, thrombosis, use of anticoagulants, tumour or cancer diseases, pregnancy, polyneuropathy, acute inflammations, and electronic and metal implants); and (8) a signed written informed consent form should be obtained. Patients who have complained of pain and poor tolerance during the ESW application were also excluded. During the treatment period, physiotherapeutic management including exercises, stretching, and physical therapy was not implemented and medications that might affect spasticity were not applied. Factors such as gender, race, and location of the stroke did not affect the qualification process. A detailed participants' recruitment procedure including both rESW intervention and measurement time points is presented in Figure 1.

#### 2.2.2. Demographic Characteristics

The subjects of the present study were 20 chronic poststroke patients (7 males and 13 females) with a mean age of 63.15 ± 12.60 years, mean body mass index (BMI) of 28.13 ± 3.49 kg/m^2^, and mean onset from stoke episode of 45.35 ± 41.12 months. All patients had ischaemic stroke and presented upper limb spasticity of the wrist and fingers flexors muscles. One group of 10 patients presented right hemiparesis and the other 10 presented left hemiparesis. The output functional activity level in the Barthel Index (BI) was set at 86.5 ± 15.1 score and the level of poststroke impairment in the National Institutes of Health Stroke Scale (NIHSS) was set at 6.20 ± 5.16 score. The clinical and demographic characteristics of patients are summarized in Table 1.

### 2.3. ESW Protocol

A rESW pneumatic device BTL-5000 ESW Power (BTL Industries Ltd., Cleveland, United Kingdom) was used to provide a single session of shock wave intervention. The pressure pulses were focused on the muscle belly of the wrist flexor hypertonic muscles of the forearm, the flexor carpi radialis (FCR), and flexor carpi ulnaris (FCU). Standard ultrasonic gel was used as a contact medium. A total of 1500 shots with a pressure of 1.5 bar and an energy flux density (EFD) of 0.030 mJ/mm^2^ were applied with the repetition frequency of shock wave irradiation of 4 pulses per second (Hz). The rESW session lasted about 6 minutes and was painless; thus, local anesthesia was not required.

### 2.4. Measurements

All measurements were performed by the same investigator immediately before (*t*_0_), immediately after (*t*_1_), 1 hour after (*t*_2_), and 24 hours after (*t*_3_) the rESW application for the Modified Ashworth Scale (MAS) and surface electromyography (sEMG), as well as immediately before (*t*_0_), 1 hour after (*t*_2_), and 24 hours after (*t*_3_) the rESW application for the infrared thermal (IRT) imaging. Measurement at *t*_1_ time point using IRT imaging was excluded from analysis due to the hypothermal effects of the USG gel used during the rESW application, which leads to significant disturbances in surface temperature distribution immediately after rESW application (this knowledge is based on the authors' experience during the pilot trials). All measurements for each patient were performed at the same time intervals and in the same sitting position, with the upper limbs lying neutrally on the rehabilitation table in a comfortable and stable position. To avoid interrater bias, all assessments were performed by the same researcher.

#### 2.4.1. The Level of Muscle Spasticity

For clinical assessment, the MAS was used to assess the level of spasticity of the radiocarpal (RC) and fingers (FF) joints. The MAS is a clinical method that tests manually the resistance during a passive movement of a joint with varying degrees of velocity using a 6-grade scale, ranging from 0 (normal muscle tone) to 4 (limb rigid in flexion or extension) [30]. A supplementary grade 1+ between the scores of 1 and 2 was used to indicate a slight increase in muscle tone, manifested by a catch, followed by minimal resistance throughout the remainder (less than half) of the range of motion. For convenience in the statistical analysis, MAS 1+ was substituted with a value of 1.5.

Additionally, the minimal clinically important difference (MCID) was applied to determine whether the rESW related alterations in the MAS scoring had clinical significance. On the basis of clinical experience and estimates reported for similar outcome measures in related literature, the MCID was set at 10% of the total range of the grades that gives 0.4 points for the MAS [31].

#### 2.4.2. The Resting Bioelectrical Activity

For the electrophysiological examination, the sEMG examination was performed to record the level of tension and bioelectrical activity of the FCR and FCU muscles under resting conditions. Dual-channel surface electromyography MyoSystem 1400L with MyoResearch XP Master Edition software (Noraxon Inc., Arizona, USA) was used to evaluate the mean values of the resting bioelectrical activity of the carpal flexor muscles.

The basic technical specification is characterized by input impedance > 100 MOhm on sEMG channels (isolated to >3000 volts); outputs of analog ±5 volts for all sEMG channels; digital 12-bit resolution per channel from USB ports; inputs of 8 sEMG channels at ±10 mV max.; 8 sensor channels at ±5 volts max.; power 100–240 VAC at 50/60 Hz (0.9 A max.); sEMG amplifier performance of 1 *µ*V sensitivity; <1 *µ*V RMS baseline noise; data acquisition of 12-bit resolution in 8 channels; USB update to PC every millisecond; high pass cut-off of 10 Hz first order on sEMG channels; low pass cut-off of selectable 500 or 1000 Hz on sEMG channels.

Resting sEMG activities of the FCR and FCU muscles were registered in stable and static conditions and each single measurement lasted 60 seconds. EMG signals were sampled at 1000 Hz and raw signals were amplified and bandpass-filtered (10–500 Hz). During data analysis and signal processing, mean values of sEMG amplitudes were calculated according to the root mean square (RMS) algorithm and the sEMG values were given in microvolts (*µ*V) unit. Full-wave rectification (FWR) was also used for all active sEMG channels, as well as the application of digital signal filtering to the elimination of background noises using a bandpass filter with a finite impulse response (FIR) for frequencies between 80 and 250 Hz.

Before the application of the electrodes, the skin was prepared with 70% alcohol to reduce skin impedance. For the sEMG recording, two pairs of self-adhesive, bipolar Ag/AgCl surface electrodes with a diameter of 10 mm and interelectrode spacing of 20 mm were used. Electrodes were placed over the centre part of the FCR and FCU muscles' bellies of the spastic upper limb. The monopolar, self-adhesive reference electrode with a diameter of 20 mm was placed on the lateral epicondyle of the humerus on the same side. Electrodes were removed after *t*_0_ measurements to perform the ESW procedure; however, they were placed at the same locations in *t*_1_, *t*_2_, and *t*_3_ measurement time points. Electrode placement was in accordance with the SENIAM (Surface Electromyography for the Noninvasive Assessment of Muscles) and ISEK (International Society of Electrophysiology and Kinesiology) recommendations for sEMG recordings [32, 33].

#### 2.4.3. The Surface Temperature Distribution

For thermographic evaluation, noninvasive and noncontact IRT imaging was conducted to detect the level of local alterations in trophic conditions connected with blood microcirculation and surface temperature distribution. Infrared thermographic images were taken with a microbolometer thermal imaging camera (MobIR M8) that works with computer software Guide IR Analyser (Wuhan Guide Infrared Co., Ltd., Hongshan District, China).

The basic technical specification is characterized by a focal plane arrays (FPA) microbolometer detector (160 × 120 pixels, 25 *μ*m) with automatic focus; a complementary metal-oxide-semiconductor (CMOS) sensor, 1600 × 1200 pixels (224 true colours); a spectral range of 8–14 *μ*m; thermal sensitivity of ≤100 mk at 30°C; a field of view and focus of 20.6°  × 15.5° and 11 mm; a temperature range of −20°C to 250°C (350°C optional); an accuracy of ±2°C or ±2% of reading; an emissivity correction of variable from 0.01 to 1.00 (in 0.01 increments).

Each patient participating in the study underwent a thorough preparation procedure of 20–30 minutes of adaptation in stable measurement conditions, cleansing the skin of creams and ointments, and the removal of potentially compressive clothing and jewellery from the area of measurement [34, 35]. IRT detection involved the region of interest of the anterior-medial surface of the forearm including the carpal flexors muscles region (CFMR).

The temperature in the research room was in the range 22–24°C with relative humidity below 45%, ensuring constant measurement conditions (no drafts and air circulation, lack of interference from devices that emit heat). The skin emissivity was set at 0.94 [36]. IRT camera has been set perpendicular to the surface of the half length of the proximal forearm of the tested spastic upper limb at a distance of 1 meter. An arithmetic mean formula was used for calculating mean, maximum, and minimum values of the surface skin temperature for all patients. The IRT values were given in Celsius degrees (°C).

### 2.5. Statistical Analysis

Statistical analysis was performed using the Statistica 10.0 software (StatSoft Poland, Dell Inc., USA). All data were shown as mean ± standard deviation (SD). To reduce a type II error and increase the power analysis, GPower 3.1.9.2 software (Microsoft Corporation, USA) was used, based on a one-way analysis of variance (ANOVA) test for a single group of dependent comparisons (power (1 − *β*) = 0.85; *α* = 0.05; effect size = 0.45), which indicated that a total sample of *n* = 20 would be needed. Normality of variable distribution was verified using the Shapiro-Wilk Test at significance level of *α* = 0.05. Intragroup comparisons for dependent variables between baseline assessment at *t*_0_ and each post-rESW measurement (*t*_1_, *t*_2_, and *t*_3_) were performed with the nonparametric Wilcoxon matched pairs test. The level of statistical significance was set at *p* < 0.05.

## 3. Results

### 3.1. The Level of Muscle Spasticity in MAS

The MAS scores for the RC joint in the baseline assessment before rESW intervention (*t*_0_) and assessments immediately after rESW (*t*_1_) and 1 hour after (*t*_2_) and 24 hours after (*t*_3_) its finalising were 1.60 ± 0.70°, 1.30 ± 0.60°, 1.50 ± 0.80°, and 1.50 ± 0.50°, respectively. Compared to the baseline, a statistically significant reduction in spasticity level in the MAS scores for the RC joint was observed in the *t*_1_ (*p* = 0.005) measurement time point only. The MAS scores also decreased in the *t*_2_ and *t*_3_ measurement time points compared to the baseline assessment; however, the differences were not statistically significant (*p* = 0.0733 and *p* = 0.0679, resp.). With respect to MCID values, the improvements in the MAS scoring for the RC joint were not exceeded (0.3 points for *t*_1_ and 0.1 points for *t*_2_ and *t*_3_).

**Figure 2:**
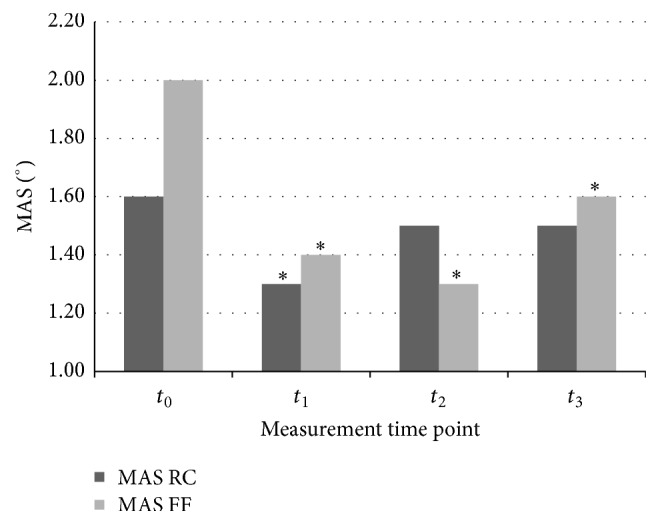
Modified Ashworth Scale (MAS) scoring of the radiocarpal (RC) joint and the fingers (FF) joints at the different time points.  ^*∗*^Statistically significant compared with baseline at *t*_0_ (*p* < 0.05).

The MAS scores for the FF joints in the baseline assessment before rESW intervention (*t*_0_) and assessments immediately after rESW (*t*_1_) and 1 hour after (*t*_2_) and 24 hours after (*t*_3_) its finalising were 2.00 ± 1.00°, 1.40 ± 0.80°, 1.30 ± 0.90°, and 1.60 ± 0.70°, respectively. Compared to the baseline, a statistically significant reduction in spasticity level in MAS scores for the FF joints was observed for all post-rESW measurement time points, namely, in *t*_1_ (*p* = 0.0007), in *t*_2_ (*p* = 0.0003), and in *t*_3_ (*p* = 0.0007) (Table 2 and Figure 2). With respect to MCID values, the improvements in the MAS scoring for the FF joints were exceeded (0.6 points for *t*_1_, 0.7 points for *t*_2_, and 0.4 points for *t*_3_).

### 3.2. The Resting Bioelectrical Activity in sEMG

Resting sEMG activity for the FCR muscle in the baseline registration before rESW intervention (*t*_0_) and assessments immediately after rESW (*t*_1_) and 1 hour after (*t*_2_) and 24 hours after (*t*_3_) its finalising were 6.47 ± 2.54 *µ*V, 4.87 ± 1.42 *µ*V, 4.84 ± 1.21 *µ*V, and 4.95 ± 1.49 *µ*V, respectively. Compared to the baseline, a statistically significant reduction in sEMG activity for the FCR muscle was observed in the *t*_1_ (*p* = 0.0303) and *t*_2_ (*p* = 0.0123) measurement time points. Resting sEMG activity registration also showed decreased tendency in the *t*_3_ measurement time point compared to the baseline; however, the difference was not statistically significant (*p* = 0.0619).

**Figure 3:**
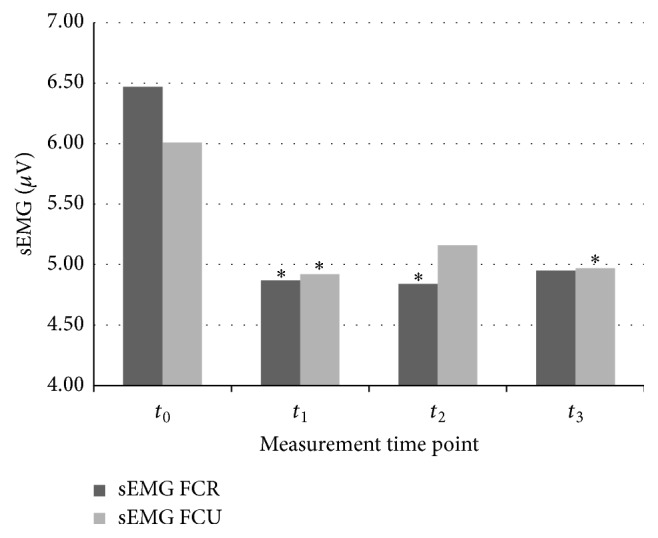
Surface electromyography (sEMG) resting activity of the flexor carpi radialis (FCR) muscle and the flexor carpi ulnaris (FCU) muscle.  ^*∗*^Statistically significant compared with baseline at *t*_0_ (*p* < 0.05).

Resting sEMG activity for the FCU muscle in the baseline registration before rESW intervention (*t*_0_) and assessments immediately after rESW (*t*_1_) and 1 hour after (*t*_2_) and 24 hours after (*t*_3_) its finalising were 6.01 ± 2.52 *µ*V, 4.92 ± 1.44 *µ*V, 5.16 ± 1.27 *µ*V, and 4.97 ± 1.39 *µ*V, respectively. Compared to the baseline, a statistically significant reduction in sEMG activity for the FCU muscle was observed in the *t*_1_ (*p* = 0.0217) and *t*_3_ (*p* = 0.0365) measurement time points. Resting sEMG activity registration also showed decreased tendency in the *t*_2_ measurement time point compared to the baseline; however, the difference was not statistically significant (*p* = 0.0627) (Table 2 and Figure 3).

### 3.3. The Surface Temperature Distribution in IRT Imaging

The IRT mean surface temperature values for the CFMR obtained in the baseline detection before rESW intervention (*t*_0_) and 1 hour after (*t*_2_) and 24 hours after (*t*_3_) its finalising were 32.13 ± 2.26°C, 32.66 ± 1.48°C, and 33.48 ± 2.07°C, respectively. Compared to the baseline, statistically significant growth in IRT imaging was reported in the *t*_3_ (*p* = 0.0441) measurement time point only. Nevertheless, the IRT temperature distribution showed a regular increased tendency in the *t*_2_ measurement time point compared to the baseline; however, the difference was not statistically significant (*p* = 0.0720).

IRT maximum surface temperature values for the CFMR obtained in the baseline detection before rESW intervention (*t*_0_) and 1 hour after (*t*_2_) and 24 hours after (*t*_3_) its finalising were 33.06 ± 2.28°C, 33.62 ± 1.55°C, and 34.54 ± 1.76°C, respectively. Compared to the baseline, statistically significant growth in IRT imaging was reported in the *t*_3_ (*p* = 0.0458) measurement time point only. Nevertheless, IRT temperature distribution showed a regular increased tendency in the *t*_2_ measurement time point compared to the baseline; however, the difference was not statistically significant (*p* = 0.4181).

**Figure 4:**
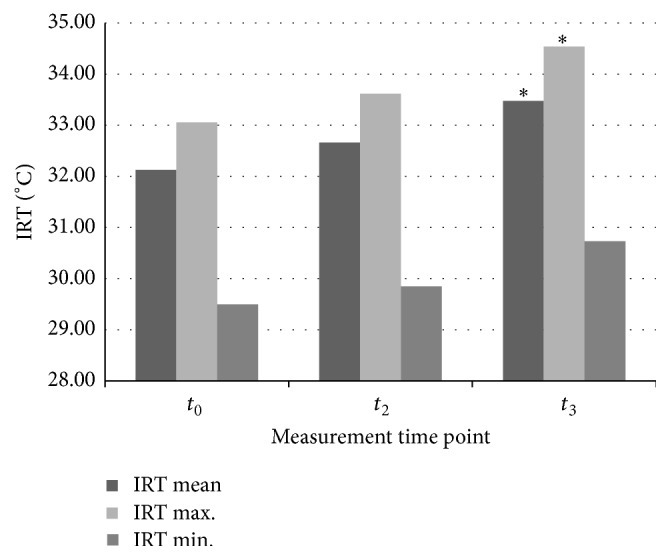
Infrared thermal (IRT) imaging camera detection of the surface temperature distribution of the carpal flexors muscles region (CFMR).  ^*∗*^Statistically significant compared with baseline at *t*_0_ (*p* < 0.05).

The IRT minimum surface temperature values for the CFMR obtained in the baseline detection before rESW intervention (*t*_0_) and 1 hour after (*t*_2_) and 24 hours after (*t*_3_) its finalising were 29.50 ± 2.29°C, 29.85 ± 2.48°C, and 30.73 ± 2.26°C, respectively. Despite a regular increased tendency in IRT temperature distribution detected in the *t*_2_ and *t*_3_ measurement time points compared to the baseline, the differences were not statistically significant (*p* = 0.5257 and *p* = 0.0930, resp.) (Table 2 and Figure 4).

## 4. Discussion

Extracorporeal shock wave is largely known as a safe and effective physical modality in the treatment of a wide range of musculoskeletal disorders including chronic pain syndromes, tendinopathies, and enthesopathies [37, 38].

Recent studies have indicated that ESW, especially in the form of focused ESW (fESW), could also be useful as a beneficial adjunctive procedure supporting the treatment of a variety of systemic disorders, such as chronic neurogenic heterotrophic ossification [39], distally symmetric polyneuropathy [40], breast cancer-related lymphoedema [41], soft tissue wounds [42], erectile dysfunction [43], bone fracture nonunions [44], femoral head osteonecrosis [45], chronic heart failure [46], coronary artery disease [47], systemic sclerosis [48], and soft tissue calcinosis [49]. The list of clinical indications for ESW treatment and subjects in research areas is continuously evolving and extending to a diverse number of disorders including muscle spasticity, especially in poststroke individuals [50].

It should be pointed out that fESW devices, which are typically generated by electromagnetic, electrohydraulic, and piezoelectric sources, are much more widespread, as much in clinical as in research usage. In the fESW type, the pressure increases rapidly from under 10 ns up to 100–1000 bar and the energy is absorbed to the level of 12 cm (the highest energy focused point of about 6 cm). On the other hand, in the rESW type, the pressure increases more slowly, up to 5 *µ*s, and reaches 1–10 bar with absorbency to the depth of 3 cm [50]. The fact is that the rESW type applied to reduce the muscle hypertonic tension caused by UMN lesion still remains under investigation to a serious extent.

This study aimed to objectively evaluate the effectiveness of a single session of rESW stimulation for wrist and fingers flexor muscles in poststroke patients with present upper limb spastic hypertonia. It was assumed that rESW application would contribute to the diminution of the resting bioelectrical activity of the spastic muscles in the sEMG recording, which would lead to an improvement of their trophic conditions and temperature distribution in the IRT imaging, as well as providing a reduction of the spasticity level in clinical evaluations using the MAS score.

It is well known that spastic muscles following stroke show progressive long-term changes in their intrinsic mechanical properties [51, 52]. Included in the most common nonneuronal consequences resulting from immobilization are muscle contractures and stiffness, muscle fiber atrophy, loss of sarcomeres, accumulation of intramuscular connective tissue, or increased fat infiltration with degeneration of mechanoreceptors [53, 54]. These degenerative changes lead to the disturbances in skeletal muscle properties, such as abnormal increased tension with inefficient contractile properties, as well as muscle atrophy which is a consequence of disturbances in blood microcirculation.

The main purpose of the sEMG examination was to determine any electrophysiological alterations in the form of diminution in the resting activity of the motor units potential (MUP), which could potentially explain the effect of the relaxation of spastic muscles under the influence of ESW application. Electrophysiological studies including a noninvasive and reliable sEMG examination play an important role in the assessment of motor neuron units activity of the spastic muscles after stroke [52]. The most common method of normalizing sEMG signals during their registration of a tested muscle is to refer them to the signal recorded from the same muscle during a maximal voluntary isometric contraction (MVIC). However, Wood et al. [55] in their systematic review summarized a biomechanical approach to spasticity as one component of UMN syndrome and confirmed that relaxation conditions could be considered for sEMG recordings in spasticity. Additionally, the RMS rectification protocol has also been used in sEMG signal registration of the spastic muscles and the results demonstrated by Albani et al. [56] showed that it may be an effective manner for detecting functional improvements and for monitoring the effects of treatment in poststroke patients. Some other researchers have also used RMS protocol in their studies with sEMG in spastic-paretic muscles [57–63].

On the other hand, the main objective of the IRT imaging was to detect a microcirculatory response and alterations in blood vessel regulation. It was assumed that rESW would contribute to an improvement in the temperature distribution of the soft tissues as a positive vascular reaction, reducing atrophy of the spastic muscles. For this purpose, a noninvasive and noncontact IRT imaging camera was used for detecting surface temperature changes in a region of interest (ROI), including the area of the tested spastic muscles [64, 65]. This is a safe and accurate tool, which is widely used in a number of diseases presenting disturbances of the skin temperature. These phenomena can be visualised as variations in a local temperature, which result from a wide range of abnormalities in the blood flow dynamics, such as soft tissue inflammation, rheumatism, enthesopathies, and injuries [66, 67]. Neves et al. [68], in their literature review, described possibilities of IRT imaging usage in neurological practice. However, there is only one clinical case report, by Nowak et al. [69], where an IRT camera was used to evaluate trophic conditions after injections of botulinum neurotoxin (BOTOX), administered directly into the spastic muscles of the upper limb. The study confirmed that treatment with BOTOX combined with physiotherapy procedures showed effective therapeutic results. An increase in the range of motion (passive and active) and a decrease in the level of muscle tone within the forearm and hand muscles in the MAS scoring were achieved, and increased local temperature due to the applied treatment was observed. Also, Ra et al. [70] conducted an evaluation with digital IRT imaging to determine muscle atrophy in patients with unilateral lumbosacral radiculopathy. They concluded that alterations in the skin temperature following lumbosacral radiculopathy were related to some clinical and MRI findings, suggesting muscle atrophy; thus IRT imaging might be useful as a complementary tool in the diagnosis of atrophy.

The obtained short-term results determined that upper limb spasticity in the MAS scores was significantly improved immediately after finalisation of the rESW application in chronic poststroke patients and a positive effect also was observed in the post-rESW measurement time point after 1 and 24 hours compared to the baseline (not significant for the RC joint).

In the electrophysiological examination during the resting sEMG recordings, a statistically significant decrease in muscle tone was observed for both the FCR and the FCU muscles immediately after the rESW session. This positive outcome was also reported 1 hour after rESW for the FCR muscle and 24 hours after rESW for the FCU muscle. It is worth noticing that the decreased tendency was kept at high level, however not significant.

On the other hand, IRT imaging results showed a regular tendency to increase temperature distribution and improve trophic conditions of the CFMR, which was increased from the measurement performed 1 hour after rESW to the measurement performed 24 hours after rESW, where the difference showed a significant level. It should be explained that the IRT measurement conducted immediately after completing the rESW session (*t*_1_) was excluded from analysis and not considered due to the significant hypothermal effect caused by the USG gel application used during the rESW application as a contact medium, which leads to significant disturbances in surface temperature distribution immediately after the rESW application. In the present study, no local anesthesia prior to rESW was implemented and adverse events following rESW application were not reported.

Among previous studies on upper limb spasticity, only Daliri et al. [22] conducted a single blind clinical study using the rESW ballistic device on wrist flexor spasticity in 15 patients after stroke. It was reported that ESW improved poststroke spasticity of the wrist flexor muscles and *α* motor neuron excitability in a neurophysiological examination (needle EMG). Positive effects in clinical assessments were observed in the follow-up analysis at 1 and 5 weeks after active rESW finalisation. Also, Kim et al. [23], in a prospective and open-label clinical study of poststroke subjects, evaluated the efficiency of pneumatic rESW in lower limb spasticity associated with plantar fasciitis where the authors observed positive results in all measured parameters, which were much greater at the *t*_2_ measurement time point, suggesting some long-term effect of rESW. These findings are very similar to our results; however, we are not able to refer to follow-up changes.

Other authors also showed a beneficial clinical effect of the fESW type in the upper [24, 26, 29] and lower limb muscles' spasticity after stroke [25, 27, 28]. However, the mechanisms behind the positive effects of ESW on spastic muscles remain unknown. Several authors [24, 27, 28], in accordance with their own neurophysiological studies using the nEMG tool, determined that the potential mechanism of ESW action is most likely linked to the reduction of muscle hypertonicity, which could be related to a direct effect improving the stiffness of connective tissue by directly acting on the rheological properties and fibrosis of the chronic spastic muscles in poststroke survivors.

Manganotti and Amelio [24] studied 20 poststroke survivors within a period not later than 9 months previously (chronic phase), where the FCR and FCU muscles were stimulated using fESW device. The authors observed no changes in either the placebo or the active ESW groups according to the nEMG parameters of motor nerve conduction (MNC). Similar results related to spinal excitability modifications after ESW application were demonstrated by Santamato et al. [27] and Sohn et al. [28].

The electrophysiological mechanisms of ESW action remain unclear, and the vascular mechanisms conditioning trophic conditions of the spastic muscles characterized by chronic atrophy are still neglected. It should be emphasized that, to the best of our knowledge, at present, no papers have observed the resting bioelectrical activity of the treated spastic muscles using the sEMG tool and the surface temperature distribution in a region of tested muscles using IRT imaging. According to these findings, it is reasonable to continue research into specialized and noninvasive tools (e.g., sEMG and IRT imaging), which allow us to precisely register some important phenomena and factors regarding the potential antispastic mechanisms following ESW treatment in spastic paresis after stroke.

*Limitations of the Study*. Our prospective, open-label, clinical trial is the third worldwide study where the rESW treatment was used to improve poststroke spasticity. Another strength of this paper was the conducting of objective sEMG and IRT assessments. However, there are some limitations to this study, for example, the small number of patients, the lack of the control group, and the lack of follow-up analysis. First of all, during the qualification procedure, 25 patients were not included in this study due to the presence of exclusion criteria; thus, the representative group has been finally collected. Additionally, in recent studies, the groups of 15–20 patients were commonly enrolled. Secondly, a control group was avoided so as to conduct an intragroup comparison only, between the pre- and post-rESW sessions. Moreover, the absence of the inclusion of a placebo group is often found in similar published studies for ethical reasons. And finally, the aim was to observe the short-term effect of a single rESW therapy at a multifaceted level including clinical outcomes, electrophysiological examination, and thermographic evaluation; thus, we decided not to analyse follow-up alterations.

## 5. Conclusion

A single session of rESW could be an effective physical treatment aimed at the reduction of upper limb spasticity and could lead to improvement of trophic conditions of the spastic muscles in poststroke survivors. Further randomized clinical studies are still required to evaluate the mechanisms of the antispastic effect of rESW, including vascular regulation and trophic conditions using IRT imaging tools.

## 6. Clinical Messages

These include the following:

- After a single session of rESW application, our results suggested positive effects on the spasticity level in chronic stroke patients.
- We observed a reduction in resting bioelectrical activity and an improvement of trophic conditions of the wrist and fingers flexor muscles.
- In the stroke patients, short-term beneficial effects were demonstrated, which lasted up to 24 hours after the rESW application.

## Figures and Tables

**Table 1:** General demographic and clinical characteristics of subjects included in the study.

Characteristic	Value
Patients (*n*)	20
Age (years)	63.15 ± 12.60
Gender (F/M)	7/13
Weight (kg)	79.50 ± 9.33
Height (m)	1.68 ± 0.07
BMI (kg/m^2^)	28.13 ± 3.49
Stroke subtype (IS/HS)	20/0
Affected site (R/L)	10/10
Time since onset (months)	45.35 ± 41.12
BI (score)	86.5 ± 15.1
NIHSS (score)	6.20 ± 5.16

BMI: body mass index; BI: Barthel Index; NIHSS: National Institutes of Health Stroke Scale; F: female; M: male; R: right; L: left; IS: ischaemic stroke; HS: haemorrhagic stroke; SD: standard deviation; *n*: number.

**Table 2:** Values of assessments for MAS scoring, resting sEMG activity, and IRT values at the different time points shown as mean ± SD.

Measurement/time points	*t*_0_	*t*_1_	*t*_2_	*t*_3_
*MAS (score)*				
RC joints	1.60 ± 0.70	1.30 ± 0.60^*∗*^	1.50 ± 0.80	1.50 ± 0.50
FF joints	2.00 ± 1.00	1.40 ± 0.80^*∗*^	1.30 ± 0.90^*∗*^	1.60 ± 0.70^*∗*^

*sEMG (µV)*				
FCR	6.47 ± 2.54	4.87 ± 1.42^*∗*^	4.84 ± 1.21^*∗*^	4.95 ± 1.49
FCU	6.01 ± 2.52	4.92 ± 1.44^*∗*^	5.16 ± 1.27	4.97 ± 1.39^*∗*^

*IRT imaging (*°*C)*				
Mean	32.13 ± 2.26	—	32.66 ± 1.48	33.48 ± 2.07^*∗*^
Max.	33.06 ± 2.28	—	33.62 ± 1.55	34.54 ± 1.76^*∗*^
Min.	29.50 ± 2.29	—	29.85 ± 2.48	30.73 ± 2.26

ESW: extracorporeal shock wave; MAS: Modified Ashworth Scale; RC: radiocarpal; FF: fingers; sEMG: surface electromyography; FCR: flexor carpi radialis; FCU: flexor carpi ulnaris; *µ*V: microvolts; IRT: infrared thermal; SD: standard deviation; *t*_0_: before ESW (baseline); *t*_1_: immediately after ESW; *t*_2_: 1 hour after ESW; *t*_3_: 24 hours after ESW.

^*∗*^Statistically significant compared with baseline at *t*_0_ (*p* < 0.05).

## References

[1] Bähr M., Frotscher M. (2012). *Duus' Topical Diagnosis in Neurology: Anatomy, Physiology, Signs, Symptoms*.

[2] Członkowska A., Niewada M., El-Baroni I. S. (2002). High early case fatality after ischaemic stroke in Poland: exploration of possible explanations in the International Stroke Trial. *Journal of the Neurological Sciences*.

[3] Sacco R. L., Kasner S. E., Broderick J. P. (2013). An updated definition of stroke for the 21st century: a statement for healthcare professionals from the American heart association/American stroke association. *Stroke*.

[4] Heuschmann P. U., Wiedmann S., Wellwood I. (2011). Three-month stroke outcome: the European Registers of Stroke (EROS) investigators. *Neurology*.

[5] Heuschmann P. U., Di Carlo A., Bejot Y. (2009). Incidence of stroke in Europe at the beginning of the 21st century. *Stroke*.

[6] Lance J., Feldmann R. G., Young R. R., Koella W. P. (1980). Symposium synopsis. *Spasticity: Disordered Motor Control*.

[7] Watkins C. L., Leathley M. J., Gregson J. M., Moore A. P., Smith T. L., Sharma A. K. (2002). Prevalence of spasticity post stroke. *Clinical Rehabilitation*.

[8] de Weerd L., Rutgers W. A. F., Groenier K. H., van der Meer K. (2011). Perceived wellbeing of patients one year post stroke in general practice—recommendations for quality aftercare. *BMC Neurology*.

[9] Yelnik A. P., Simon O., Parratte B., Gracies J. M. (2010). How to clinically assess and treat muscle overactivity in spastic paresis. *Journal of Rehabilitation Medicine*.

[10] Brainin M., Norrving B., Sunnerhagen K. S. (2011). Poststroke chronic disease management: towards improved identification and interventions for poststroke spasticity-related complications. *International Journal of Stroke*.

[11] Malhotra S., Pandyan A. D., Rosewilliam S., Roffe C., Hermens H. (2011). Spasticity and contractures at the wrist after stroke: time course of development and their association with functional recovery of the upper limb. *Clinical Rehabilitation*.

[12] Bhimani R. H., Anderson L. C., Henly S. J., Stoddard S. A. (2011). Clinical measurement of limb spasticity in adults: state of the science. *Journal of Neuroscience Nursing*.

[13] Marciniak C. (2011). Poststroke hypertonicity: upper limb assessment and treatment. *Topics in Stroke Rehabilitation*.

[14] Smania N., Picelli A., Munari D. (2010). Rehabilitation procedures in the management of spasticity. *European Journal of Physical and Rehabilitation Medicine*.

[15] Veerbeek J. M., van Wegen E., van Peppen R. (2014). What is the evidence for physical therapy poststroke? A systematic review and meta-analysis. *PLoS ONE*.

[16] Lee J.-Y., Kim S.-N., Lee I.-S., Jung H., Lee K.-S., Koh S.-E. (2014). Effects of extracorporeal shock wave therapy on spasticity in patients after brain injury: a meta-analysis. *Journal of Physical Therapy Science*.

[17] Mori L., Marinelli L., Pelosin E. (2014). Shock waves in the treatment of muscle hypertonia and dystonia. *BioMed Research International*.

[18] Amelio E., Manganotti P. (2010). Effect of shock wave stimulation on hypertonic plantar flexor muscles in patients with cerebral palsy: a placebo-controlled study. *Journal of Rehabilitation Medicine*.

[19] El-Shamy S. M., Eid M. A., El-Banna M. F. (2014). Effect of extracorporeal shock wave therapy on gait pattern in hemiplegic cerebral palsy: a randomized controlled trial. *American Journal of Physical Medicine and Rehabilitation*.

[20] Gonkova M. I., Ilieva E. M., Ferriero G., Chavdarov I. (2013). Effect of radial shock wave therapy on muscle spasticity in children with cerebral palsy. *International Journal of Rehabilitation Research*.

[21] Marinelli L. M., Mori L., Solaro C. (2015). Effect of radial shock wave therapy on pain and muscle hypertonia: a double-blind study in patients with multiple sclerosis. *Multiple Sclerosis*.

[22] Daliri S. S., Forogh B., Emami Razavi S. Z., Ahadi T., Madjlesi F., Ansari N. N. (2015). A single blind, clinical trial to investigate the effects of a single session extracorporeal shock wave therapy on wrist flexor spasticity after stroke. *NeuroRehabilitation*.

[23] Kim T. G., Bae S. H., Kim G. Y., Kim K. Y. (2015). The effects of extracorporeal shock wave therapy on stroke patients with plantar fasciitis. *Journal of Physical Therapy Science*.

[24] Manganotti P., Amelio E. (2005). Long-term effect of shock wave therapy on upper limb hypertonia in patients affected by stroke. *Stroke*.

[25] Moon S. W., Kim J. H., Jung M. J. (2013). The effect of extracorporeal shock wave therapy on lower limb spasticity in subacute stroke patients. *Annals of Rehabilitation Medicine*.

[26] Santamato A., Notarnicola A., Panza F. (2013). SBOTE study: extracorporeal shock wave therapy versus electrical stimulation after botulinum toxin type a injection for post-stroke spasticity-a prospective randomized trial. *Ultrasound in Medicine and Biology*.

[27] Santamato A., Micello M. F., Panza F. (2014). Extracorporeal shock wave therapy for the treatment of poststroke plantar-flexor muscles spasticity: a prospective open-label study. *Topics in Stroke Rehabilitation*.

[28] Sohn M. K., Cho K. H., Kim Y., Hwang S. L. (2011). Spasticity and electrophysiologic changes after extracorporeal shock wave therapy on gastrocnemius. *Annals of Rehabilitation Medicine*.

[29] Troncati F., Paci M., Myftari T., Lombardi B. (2013). Extracorporeal Shock Wave Therapy reduces upper limb spasticity and improves motricity in patients with chronic hemiplegia: a case series. *NeuroRehabilitation*.

[30] Bohannon R. W., Smith M. B. (1987). Interrater reliability of a modified Ashworth scale of muscle spasticity. *Physical Therapy*.

[31] Song R., Tong K.-Y., Hu X., Zhou W. (2013). Myoelectrically controlled wrist robot for stroke rehabilitation. *Journal of NeuroEngineering and Rehabilitation*.

[32] De Luca C. J., Gilmore L. D., Kuznetsov M., Roy S. H. (2010). Filtering the surface EMG signal: movement artifact and baseline noise contamination. *Journal of Biomechanics*.

[33] Staudenmann D., Roeleveld K., Stegeman D. F., van Dieen J. H. (2010). Methodological aspects of SEMG recordings for force estimation—a tutorial and review. *Journal of Electromyography and Kinesiology*.

[34] Nahm F. S. (2013). Infrared thermography in pain medicine. *Korean Journal of Pain*.

[35] Pereira C. B., Czaplik M., Blanik N., Rossaint R., Blazek V., Leonhardt S. (2014). Contact-free monitoring of circulation and perfusion dynamics based on the analysis of thermal imagery. *Biomedical Optics Express*.

[36] Diakides N. A., Bronzino J. D. (2008). *Medical Infrared Imaging*.

[37] Speed C. (2014). A systematic review of shockwave therapies in soft tissue conditions: focusing on the evidence. *British Journal of Sports Medicine*.

[38] Dymarek R., Bidzińska G., Zwierzchowski K., Słupska L., Ptaszkowski K., Halski T. (2015). Evaluation of the effectiveness of extracorporeal shock wave therapy in selected musculoskeletal system disorders of the inflammatory etiology—a critical review of the literature. *Wiadomości Lekarskie*.

[39] Choi Y. M., Hong S. H., Lee C. H., Kang J. H., Oh J. S. (2015). Extracorporeal shock wave therapy for painful chronic neurogenic heterotopic ossification after traumatic brain injury: a case report. *Annals of Rehabilitation Medicine*.

[40] Lohse-Busch H., Reime U., Falland R. (2014). Symptomatic treatment of unresponsive wakefulness syndrome with transcranially focused extracorporeal shock waves. *NeuroRehabilitation*.

[41] Kim S.-Y., Bae H., Ji H. M. (2015). Computed tomography as an objective measurement tool for secondary lymphedema treated with extracorporeal shock wave therapy. *Annals of Rehabilitation Medicine*.

[42] Dymarek R., Halski T., Ptaszkowski K., Slupska L., Rosinczuk J., Taradaj J. (2014). Extracorporeal shock wave therapy as an adjunct wound treatment: a systematic review of the literature. *Ostomy Wound Management*.

[43] Vardi Y., Appel B., Kilchevsky A., Gruenwald I. (2012). Does low intensity extracorporeal shock wave therapy have a physiological effect on erectile function? Short-term results of a randomized, double-blind, sham controlled study. *Journal of Urology*.

[44] Vulpiani M. C., Vetrano M., Conforti F. (2012). Effects of extracorporeal shock wave therapy on fracture nonunions. *American Journal of Orthopedics (Belle Mead, N.J.)*.

[45] Wang C.-J., Cheng J.-H., Huang C.-C., Yip H.-K., Russo S. (2015). Extracorporeal shockwave therapy for avascular necrosis of femoral head. *International Journal of Surgery*.

[46] Assmus B., Walter D. H., Seeger F. H. (2013). Effect of shock wave-facilitated intracoronary cell therapy on LVEF in patients with chronic heart failure: the CELLWAVE randomized clinical trial. *The Journal of the American Medical Association*.

[47] Yang P., Guo T., Wang W. (2013). Randomized and double-blind controlled clinical trial of extracorporeal cardiac shock wave therapy for coronary heart disease. *Heart and Vessels*.

[48] Tinazzi E., Amelio E., Marangoni E. (2011). Effects of shock wave therapy in the skin of patients with progressive systemic sclerosis: a pilot study. *Rheumatology International*.

[49] Sultan-Bichat N., Menard J., Perceau G., Staerman F., Bernard P., Reguiaï Z. (2012). Treatment of calcinosis cutis by extracorporeal shock-wave lithotripsy. *Journal of the American Academy of Dermatology*.

[50] Dymarek R., Ptaszkowski K., Słupska L., Halski T., Taradaj J., Rosińczuk J. (2016). Effects of extracorporeal shock wave on upper and lower limb spasticity in post-stroke patients: a narrative review. *Topics in Stroke Rehabilitation*.

[51] Gao F., Zhang L.-Q. (2008). Altered contractile properties of the gastrocnemius muscle poststroke. *Journal of Applied Physiology*.

[52] Li X., Wang Y.-C., Suresh N. L., Rymer W. Z., Zhou P. (2011). Motor unit number reductions in paretic muscles of stroke survivors. *IEEE Transactions on Information Technology in Biomedicine*.

[53] Gracies J.-M. (2005). Pathophysiology of spastic paresis. I: paresis and soft tissue changes. *Muscle and Nerve*.

[54] Lieber R. L., Steinman S., Barash I. A., Chambers H. (2004). Structural and functional changes in spastic skeletal muscle. *Muscle and Nerve*.

[55] Wood D. E., Burridge J. H., van Wijck F. M. (2005). Biomechanical approaches applied to the lower and upper limb for the measurement of spasticity: a systematic review of the literature. *Disability and Rehabilitation*.

[56] Albani G., Cimolin V., Galli M. (2010). Use of surface EMG for evaluation of upper limb spasticity during botulinum toxin therapy in stroke patients. *Functional Neurology*.

[57] Bhakta B. B., Cozens J. A., Chamberlain M. A., Bamford J. M. (2001). Quantifying associated reactions in the paretic arm in stroke and their relationship to spasticity. *Clinical Rehabilitation*.

[58] Kuriki H. U., de Azevedo R. N., de Carvalho A. C., de Azevedo F. M., Negrão-Filho R. F., Alves N. (2010). The surface electromyography analysis of the non-plegic upper limb of hemiplegic subjects. *Arquivos de Neuro-Psiquiatria*.

[59] Doğan-Aslan M., Nakipolu-Yüzer G. F., Doan A., Karabay I., Özgirgin N. (2012). The effect of electromyographic biofeedback treatment in improving upper extremity functioning of patients with hemiplegic stroke. *Journal of Stroke and Cerebrovascular Diseases*.

[60] Fleuren J. F. M., Snoek G. J., Voerman G. E., Hermens H. J. (2009). Muscle activation patterns of knee flexors and extensors during passive and active movement of the spastic lower limb in chronic stroke patients. *Journal of Electromyography and Kinesiology*.

[61] Sin M., Kim W.-S., Park D. (2014). Electromyographic analysis of upper limb muscles during standardized isotonic and isokinetic robotic exercise of spastic elbow in patients with stroke. *Journal of Electromyography and Kinesiology*.

[62] Boudarham J., Roche N., Teixeira M. (2014). Relationship between neuromuscular fatigue and spasticity in chronic stroke patients: a pilot study. *Journal of Electromyography and Kinesiology*.

[63] Mazzaro N., Nielsen J. F., Grey M. J., Sinkjaer T. (2007). Decreased contribution from afferent feedback to the soleus muscle during walking in patients with spastic stroke. *Journal of Stroke and Cerebrovascular Diseases*.

[64] Allen J., Howell K. (2014). Microvascular imaging: techniques and opportunities for clinical physiological measurements. *Physiological Measurement*.

[65] Tattersall G. J. (2016). Infrared thermography: a non-invasive window into thermal physiology. *Comparative Biochemistry and Physiology Part A: Molecular & Integrative Physiology*.

[66] Ring E. F. J., Ammer K. (2012). Infrared thermal imaging in medicine. *Physiological Measurement*.

[67] Sanchis-Sánchez E., Vergara-Hernández C., Cibrián R. M., Salvador R., Sanchis E., Codoñer-Franch P. (2014). Infrared thermal imaging in the diagnosis of musculoskeletal injuries: a systematic review and meta-analysis. *American Journal of Roentgenology*.

[68] Neves E. B., Vilaça-Alves J., Rosa C., Reis V. M. (2015). Thermography in neurologic practice. *Open Neurology Journal*.

[69] Nowak I., Mraz M., Mraz M. (2013). Thermovision evaluation of spastic upper limb of the post-stroke patient treated with botulinum toxin—a case report. *Acta Bio-Optica et Informatica Medica*.

[70] Ra J. Y., An S., Lee G.-H., Kim T. U., Lee S. J., Hyun J. K. (2013). Skin temperature changes in patients with unilateral lumbosacral radiculopathy. *Annals of Rehabilitation Medicine*.

